# Interferon-Gamma-Mediated Osteoimmunology

**DOI:** 10.3389/fimmu.2018.01508

**Published:** 2018-06-29

**Authors:** Mengjia Tang, Li Tian, Guojing Luo, Xijie Yu

**Affiliations:** Laboratory of Endocrinology and Metabolism, Department of Endocrinology and Metabolism, National Key Laboratory of Biotherapy/Collaborative Innovation Center of Biotherapy and Cancer Center, West China Hospital, Sichuan University, Chengdu, Sichuan, China

**Keywords:** osteoimmunology, interferon-gamma, osteoclasts, osteoblasts, rheumatoid arthritis, postmenopausal osteoporosis, human immunodeficiency virus

## Abstract

Osteoimmunology is the interdiscipline that focuses on the relationship between the skeletal and immune systems. They are interconnected by shared signal pathways and cytokines. Interferon-gamma (IFN-γ) plays important roles in immune responses and bone metabolism. IFN-γ enhances macrophage activation and antigen presentation. It regulates antiviral and antibacterial immunity as well as signal transduction. IFN-γ can promote osteoblast differentiation and inhibit bone marrow adipocyte formation. IFN-γ plays dual role in osteoclasts depending on its stage. Furthermore, IFN-γ is an important pathogenetic factor in some immune-mediated bone diseases including rheumatoid arthritis, postmenopausal osteoporosis, and acquired immunodeficiency syndrome. This review will discuss the contradictory findings of IFN-γ in osteoimmunology and its clinical application potential.

## Introduction

Osteoimmunology is the interdiscipline that focuses on the relationship between the skeletal and immune systems. In 2000, Arron and Choi first proposed the term “osteoimmunology” (1). Over the last 20 years, accumulating evidence has shown the involvement of the skeletal system in the process of hematoiesis and its influences in the immune system. Simultaneously, multiple immune cells and immune-derived cytokines are involved in bone homeostasis. The complex crossover between the skeletal and immune systems can also be found in various clinically relevant diseases such as osteoporosis, rheumatoid arthritis (RA), ankylosing spondylitis, human immunodeficiency virus (HIV), inflammatory bowel disease, and systemic lupus erythematosus (2–5). With a better understanding of osteoimmunology, there is higher possibility to prevent and treat immune-mediated bone diseases by targeting immune cells or immune-derived cytokines. Interferon-gamma (IFN-γ) is one of the immune-derived cytokines in the innate and adaptive immune responses. Recent studies have showed the active roles of IFN-γ in the differentiation of osteoclasts, osteoblasts, and bone marrow adipocytes. However, the effect of IFN-γ in osteoimmunology is less explored. This review will summarize current understanding in this field and discuss the clinical application value of IFN-γ in osteoimmunology.

## Osteoimmunology: Interaction Between the Skeletal and Immune Systems

**Figure 1:**
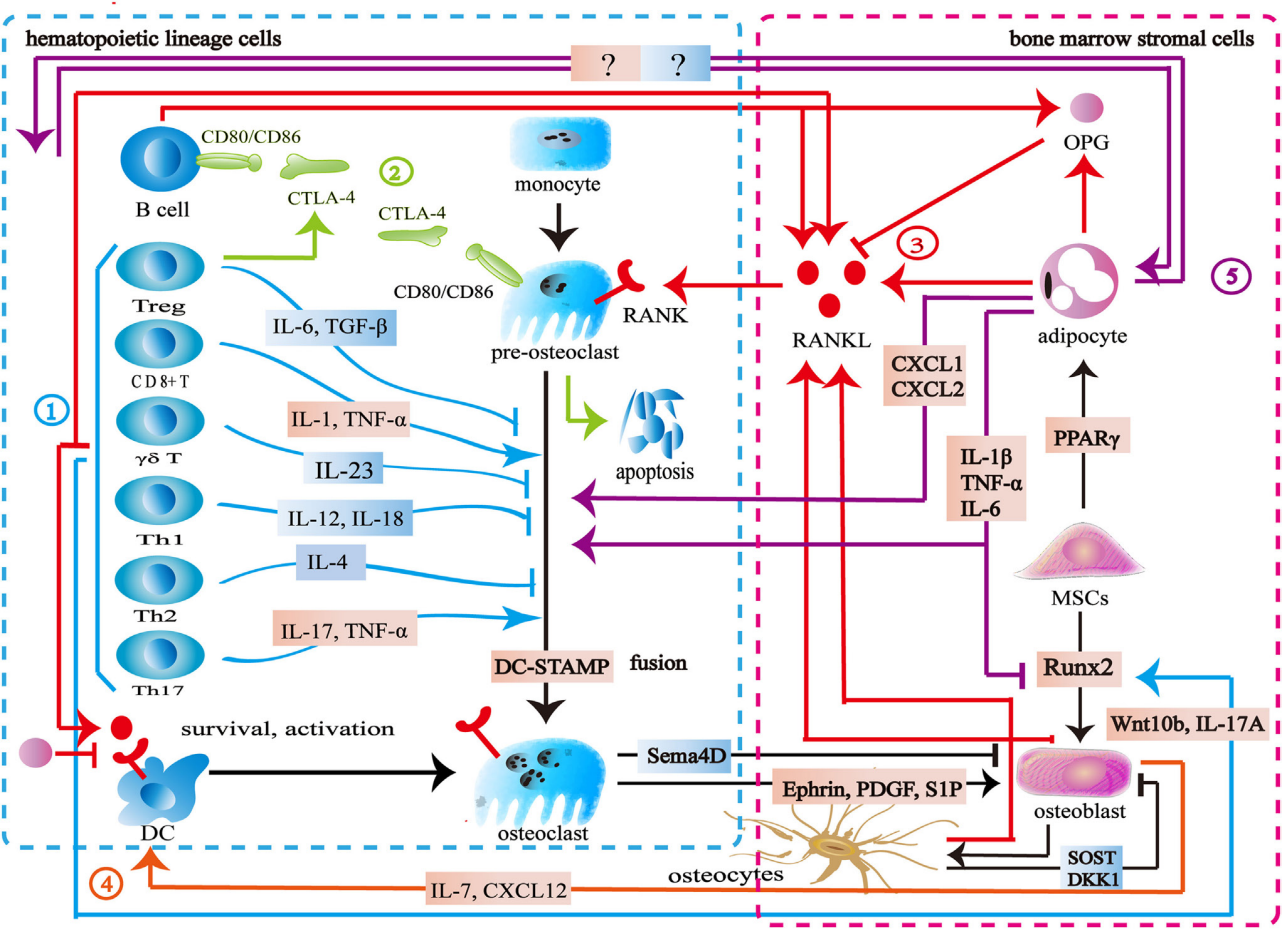
Interaction between the skeletal and immune systems. ① The immune cells produce various cytokines and molecules to regulate bone cells. IL-23, IL-6, IL-12, IL-18, IL-4, interferon-gamma (IFN-γ), TGF-β, and OPG inhibit osteoclastogenesis, while IL-1, IL-6, IL-17, TNF-α, TNF-β, RANKL, and macrophage colony stimulating factor promote osteoclast formation. Wnt10b, IL-17A, and BMP-2 enhance osteoblast formation. ② Cytotoxic T lymphocyte antigen 4 (CTLA-4) promotes the apoptosis of osteoclast precursors by binding to CD80/CD86 on the surface of osteoclasts to induce indoleamine 2,3-dioxygenase. CTLA-4 binds to CD80/CD86 on the surface of B cells to inhibit antigen presentation and the costimulatory signaling in T cells. ③ The skeletal and immune systems share RANK–RANKL–OPG pathways. ④ Osteoblasts produce CXCL12 and IL-7 to maintain lymphopoiesis. ⑤ Bone marrow adipocytes produce several factors to regulate osteoclasts, osteoblasts, and immune cells.

The immune system consists of immune organs, multiple immune cells, and immune factors, which participate in the process of immune defense, immune surveillance, and immune homeostasis. Bone cells including osteoclasts, osteoblasts, bone lining cells, and osteocytes are indispensable moderators for bone homeostasis. Bone provides physiological function for life by maintaining a balance between bone resorption and bone formation. The skeletal and immune systems are interconnected by shared signal pathways and intermediary cytokines (Figure 1).

Bone marrow is both a hematopoietic organ and an immune organ. In the bone marrow microenvironment, hematopoietic stem cells differentiate into erythrocytes, granulocytes, platelets, and all kinds of immune cells including monocytes, dendritic cells, T cells, B cells, and natural killer (NK) cells (6, 7). Among them, the monocytes/macrophages from hematopoietic lineage are osteoclast precursors (8, 9). Furthermore, osteoblasts are derived from mesenchymal stem cells (MSCs) in the bone marrow (10). Bone marrow is located in relatively confined bone marrow cavity. Several diversified immune cells including CD4^+^ T cells, CD8^+^ T cells, Treg cells, B cells, dendritic cells, and NK cells reside in the bone marrow (11–21). Osteoclasts and osteoblasts attach to the bone surface. Osteocytes are embed in the bone matrix. Bone cells thus share the same microenvironment with immune cells in the bone marrow (Figure 1).

Similar to immune cells, osteoclasts originate from multipotent hematopoietic stem cells. Macrophage colony stimulating factor (M-CSF) binds to c-Fms to mediate the cytoskeletal rearrangement, survival, and proliferation of osteoclasts through the Grb2/extracellular signal-regulated kinase (ERK) and Src/PI3K/Akt pathways (22–25). Furthermore, M-CSF activates PU.1 and MITF to initiate early osteoclast differentiation (26). After that, the RANK–RANKL pathway regulates osteoclast differentiation. RANKL binds to RANK on osteoclast precursors and recruits tumor necrosis factor receptor-associated factors 6 (TRAF6) to activate the mitogen-activated protein kinase (MAPK) family and IkB kinase (27). The MAPK family includes ERK, c-Jun N-terminal kinase (JNK), and p38 MAPK (28, 29). The downstream signaling NF-κB and AP-1 regulate the expression of NFATc1 to promote osteoclast formation (30–33). TREM2 and OSCAR, immunoglobulin-like receptors on the surface of osteoclasts, are associated with immunoreceptor tyrosine-based activation motif (ITAM)-harboring adapters, FcRγ and DAP12, to initiate RANK–RANKL costimulatory signaling. The signaling pathways increase intracellular calcium levels and then regulate transcription of NFATc1 (34–36). Another important member of bone homeostasis is the osteoblasts derived from MSCs. Bone morphogenetic protein and Wnt signaling are responsible for the differentiating of MSCs toward the osteoprogenitor lineage (37, 38). Runt-related transcription factor 2 (Runx2) and osterix (Osx) play crucial roles in this process (39, 40). Finally, some osteoblasts can become osteocytes (41) (Figure 1).

As mentioned above, many signaling pathways are involved in regulating the formation of osteoblasts, osteocytes, and osteoclasts. Among them, the RANK–RANKL–OPG is the most important shared signaling pathway between the skeletal and immune systems. RANKL and RANK are expressed not only by the skeletal system but also by the immune system. In addition to osteoblasts and osteocytes, activated T cells, B cells, NK cells, fibroblasts, and thymus cells can also produce RANKL. Apart from osteoclasts and their progenitor cells, dendritic cells also express RANK (42). Previous studies suggested that osteoblasts were the main source of OPG. New findings show that B cells are also a source of OPG (43, 44). The RANK–RANKL pathway participates in the development of lymph nodes, the self-tolerance of T cells, the survival and apoptosis of dendritic cells, and antigen presentation (45, 46). As an antagonist of RANKL, OPG reduces T cell activation by antagonistic RANK–RANKL. It also directly inhibits the survival and function of dendritic cells (46, 47). In addition to its role as the downstream of the costimulatory signal of RANK–RANKL, ITAM also participates in various signaling cascade in the immune process. Adapter protein DAP12 and FcRγ with ITAM exist in a variety of immune cells including NK cells, neutrophils, macrophages, dendritic cells, basophils, eosinophilic cells, and mast cells to regulate immune response (48). Cytotoxic T lymphocyte antigen 4 (CTLA-4) binds to CD80/CD86 on the surface of the antigen-presenting cells to inhibit the costimulatory signaling of T cells. By binding to CD80/CD86 on the surface of osteoclasts and inducing indoleamine 2,3-dioxygenase (IDO), CTLA-4 promotes osteoclast precursor apoptosis (49). Moreover, Eph receptor, CD100, and osteopontin have the capacity of regulating both the skeletal and immune systems (50–56) (Figure 1).

A variety of immune cells including T cells, B cells, NK cells, and dendritic cells produce various kinds of cytokines to regulate bone metabolism. Activated CD4^+^T cells and CD8^+^ T cells secrete RANKL, TNF-α, IL-1, IL-6, and IL-17 to promote osteoclast formation. Th17 cells secrete IL-17, RANKL, TNF-α, IL-1, and IL-6 to induce osteoclast formation (57, 58). γδT cells secrete IL-23 to promote osteoclast formation (59, 60). Treg cells inhibit osteoclast formation directly through CTLA-4 and indirectly through TGF-β, IL-4, and IL-10 (57). FoxP3^+^ CD8 T-cells produce IFN-γ and CTLA-4 to inhibit osteoclastogenesis while RANKL to promote osteoclastogenesis (61). Both Th1 and Th2 cells secrete IFN-γ and IL-4 to inhibit osteoclast formation (62–64). Furthermore, IL-23 and IL-6 could inhibit osteoclast formation (65, 66). B cells produce TNF-α and TNF-β to promote osteoclast formation, while producing OPG, IFN-γ, and TGF-β to inhibit osteoclast formation (67–69). NK cells play an active role in osteoclast formation by producing RANKL and M-CSF (70). Apart from regulating osteoclasts, some immune cells produce cytokines to regulate osteoblast formation. CD8^+^ T cells produce Wnt10b to activate Wnt signaling while γδ T cells secrete IL-17A to promote bone formation (59, 71). Furthermore, multiple bone cells are capable of influencing the immune system. Osteoblasts regulate B cell development by expressing CXCL12 and IL-7 to influence the common lymphoid progenitors (72). DLL4 derived from osteoblasts promotes T-linage competent cell function (73). Osteocytes are involved in lymphopoiesis by maintaining primary lymphoid organ microenvironment (74) (Figure 1).

The interaction between bone cells and immune cells is acknowledged in osteoimmunology. However, the importance of bone marrow adipocytes in osteoimmunology seems to be ignored. Bone marrow adipocytes and osteoblasts share the same origin. Adipogenic transcription factors, PPARγ, and C/EBPs promote MSCs to differentiate into adipocytes in the bone marrow (75). As an important component of bone marrow, bone marrow adipocytes share a common microenvironment with bone cells and immune cells. Bone marrow adipocytes produce OPG and RANKL to regulate the RANK–RANKL–OPG pathway and osteoimmunology (76–78). In puberty, bone marrow adipocytes seem to be beneficial to the skeletal system, whereas the increase of bone marrow adipocytes is associated with bone loss in aging, starvation, osteoporosis, unloading, and type 1 diabetes (79). In these pathological states, bone marrow adipocytes produce several factors including M-CSF, IL-6, IL-1 β, TNF-α, CXCL1, and CXCL2 to disturb the balance between the skeletal and immune systems (77, 80). The exact mechanism remains unknown, however, this imbalance is hypothesized to increase osteoclastogenesis and reduce osteogenesis (81). Furthermore, CXCL1 and CXCL2 can activate osteoclasts (82). Further studies are needed to address the physiopathological roles among bone cells, immune cells, and bone marrow adipocytes in osteoimmunology (Figure 1).

Osteoimmunology is involved in immune-mediated bone diseases. Menopause and aging are pathogenesis factors for osteoporosis. An increasing number of studies have shown that immune activation and bone loss concomitantly initiate during aging and menopause (4, 52, 83). Glucocorticoids have inhibitory effects on a broad range of immune responses. Their clinical application may cause osteoporosis. The immunosuppression and bone loss may be correlated (84). In RA, activated immune cells (T cells and B cells) and upregulation of some immune factors, e.g., TNF-α and RANKL result in bone destruction and bone loss (52). Local bone erosion and hyperplasia coexist in ankylosing spondylitis. Th17, TNF-α, IL-23, and IL-17 are involved in the pathological process of ankylosing spondylitis (85). In addition, immune deficiency also has detrimental effects on bone health. Patients with acquired immunodeficiency syndrome have lower bone mineral density and a higher fracture risk. Activated B cells and T cells as well as various immune factors may be the reason behind this (86, 87). The imbalance of bone resorption and bone formation caused by immune dysfunction provides a clue to seek therapeutic targets through osteoimmunology.

Osteoimmunology emphasizes the interaction between the skeletal and the immune systems. The source of IFN-γ is not as widespread as TNF-α, IL-6 and more limited to the immune system (88). Several studies also showed that IFN-γ was involved in bone homeostasis. The involvement of IFN-γ in cancer, atherosclerosis, and hematopoiesis has already been summarized (89–92). However, the role of IFN-γ in osteoimmunology has not been reviewed in detail.

## IFN-γ in Osteoimmunology

### Signaling Pathway and Function of IFN-γ

Components of the interferon family include type I IFN, type II IFN, and type III IFN. Type I IFN and type III IFN contain several sub-types whereas IFN-γ is the only type II interferon. IFN-γ is produced by multiple types of lymphocyte cells including CD4^+^ and CD8^+^ T cells, Treg cells, FoxP3^+^ CD8 T-cells, γδ T cells, B cells, and NK cells (61, 93). Apart from lymphocyte cells, other immune cells are also capable of producing IFN-γ including monocyte/macrophage, dendritic cells, and neutrophile granulocytes (94–97). Abundant evidence suggests that T cells and NK cells are the major sources of IFN-γ (98). MSCs secrete low-level IFN-γ to regulate hematopoiesis (99). Early studies demonstrated that the monocyte/macrophage and dendritic cells produced IL-12 and IL-18 to upregulate IFN-γ in the monocyte/macrophage, dendritic cells, NK cells, and Th1 cells. IFN-γ also upregulates its own expression in the macrophages by autocrine (96). Through cell–cell contact and factors existing in the co-culture medium, MSCs facilitate IL-12/IL-18-stimulated NK cells to secrete IFN-γ (100). Furthermore, IL-4 and IL-10 negatively regulate its expression (96). In a word, a variety of immune cells produce IFN-γ and the activity and quantity of IFN-γ are dependent on certain inflammatory and immune states.

**Figure 2:**
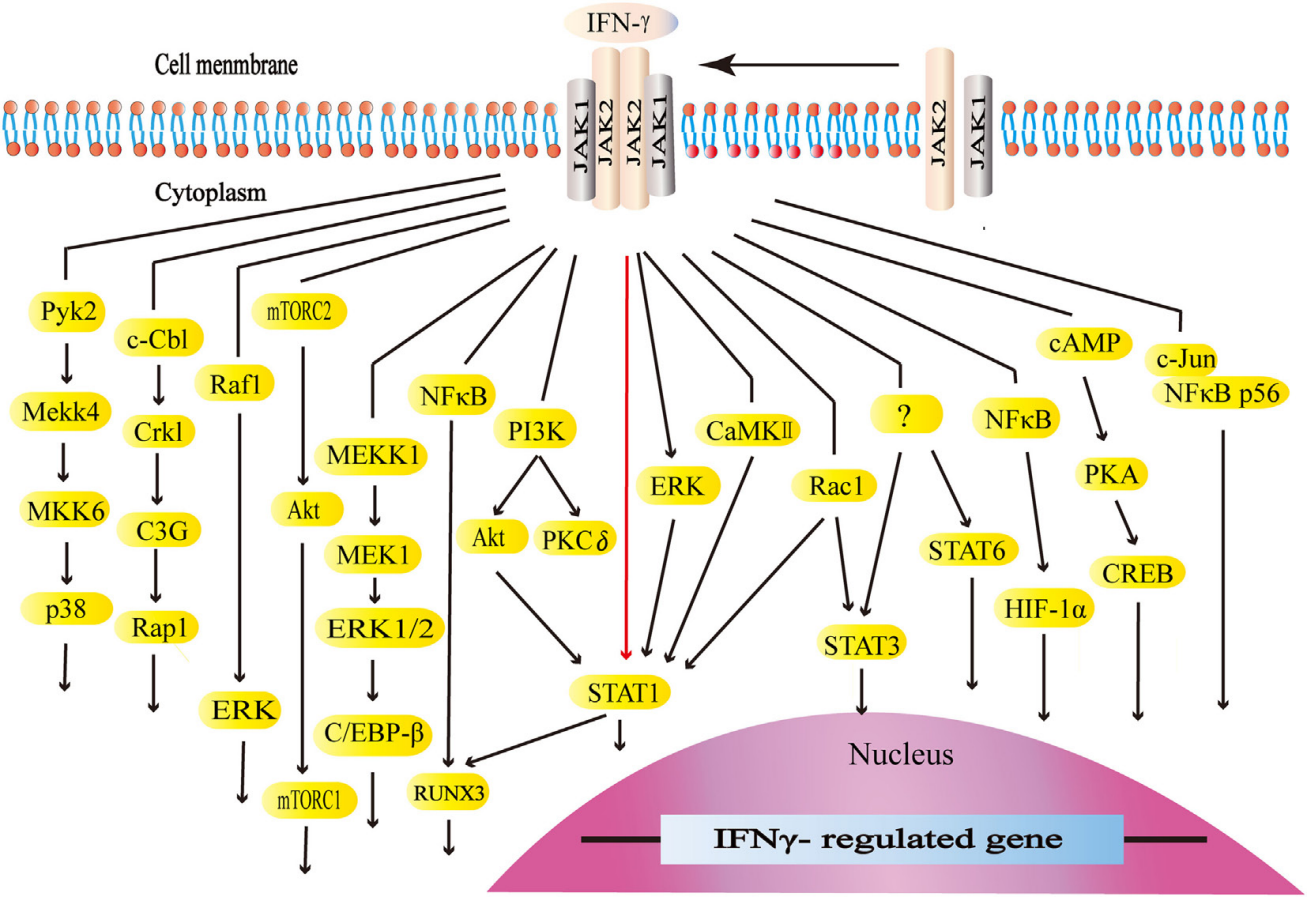
The canonical and non-canonical signaling pathways of interferon-gamma (IFN-γ). The JAK–STAT1 is the canonical signaling pathway. Apart from JAK–STAT1, IFN-γ activates other downstream factors. IFN-γ activates the kinase like PI3K, Pyk2, extracellular signal-regulated kinase (ERK), and CaMKII as well as GTPases like Raf1 and Rac1 to initiate multiple downstream signaling cascades. IFN-γ stimulates E3 ubiquitin-protein ligase c-Cbl, NF-κB, c-Jun, and cAMP, which are also involved in multiple pathways.

Interferon-gamma interacts with its receptors to trigger the cellular responses. IFN-γ receptors are ubiquitously expressed on the cellular surface of nearly all types of cells except for erythrocytes (101, 102). The functional receptors consist of receptor α chain (IFNGR1) and β chain (IFNGR2), both of which are vital for activating IFN-γ signaling (101–103). The expression of IFNGR1 seems to be constitutive. On the other hand, the expression of IFNGR2 is inducible because of its extremely low level at typical states. IFNGR1 is required for ligand binding with IFN-γ binding sets in the extracellular domain. Furthermore, IFNGR1 is critical for the downstream signal transduction with the tyrosine kinase JAK1 binding sets and STAT1 binding sets. IFNGR2 contains the tyrosine kinase JAK2 binding sets (101). The JAK–STAT1 pathway is the canonical signaling pathway of IFN-γ and commonly exists in many intracellular responses. By binding to receptors, IFN-γ activates JAK2 and JAK1. The formation of STAT1 binding sets leads to STAT1 recruitment, activation, and homodimer formation. STAT1 homodimers translocate to the nucleus and bind to the gamma-interferon activation site, the promoter regions of interferon-stimulated genes (ISGs), to initiate or turn off its expression (101, 104). ISGs play a vital role in the subsequent biological process (105). Apart from the canonical JAK–STAT1 pathway, IFN-γ also initiates the MAP kinase pathway, PI3K pathway, and NF-κB pathway. Meanwhile, IFN-γ could activate transcription factors STAT3, STAT5, and AP-1. The canonical and non-canonical signaling pathways form its complex signal network (106–128) (Figure 2).

Interferon-gamma participates in many immunological functions (innate and adaptive immune responses) and inflammation. IFN-γ enhances macrophage activation and antigen presentation. It is broadly involved in antiviral and antibacterial immunity as well as signal transduction. Due to the complex role of IFN-γ in physiological and pathological conditions, it is difficult to label its effect as pro-inflammatory or anti-inflammatory (98). Many studies aim to explore the possibility of IFN-γ as a drug to treat certain types of cancer, idiopathic pulmonary fibrosis, invasive fungal infection, hepatic fibrosis, RA, and mycosis fungoides (129–134). The US Food and Drug Administration has approved IFN-γ-1b (marketed as Actimmune) to decrease the number and severity of infections in patients with chronic granulomatous disease and to delay the progression of severe, malignant osteopetrosis.

### Effect of IFN-γ on the Skeletal System

#### Effects of IFN-γ on Osteoblasts

Ruiz et al. reported that human osteoblast-like cells express a slight level of IFN-γ (135). Early studies showed that IFN-γ increases alkaline phosphatase (ALP) activity in osteoblast-like cells derived from human femur (136). Meanwhile, IFN-γ promotes calvarial osteoblast formation by upregulating osteogenic factors including Runx2, osterix, Alp, and osteocalcin (137). Activated CD4^+^ T cells can promote human MSCs to differentiate into osteoblasts in the co-culture system. After blocking IFN-γ with antibodies, osteogenic activity is downregulated (138). Human MSCs also produce IFN-γ to promote its own osteogenic differentiation. The upregulated osteogenic transcription factors Runx2 is responsible for osteoblast formation (139). MSCs are able to differentiate into bone marrow adipocytes as well as osteoblasts. IFN-γ inhibits MSCs to differentiate into adipocytes through the downregulation of adipogenic transcription factor PPARγ and the upregulation of osteogenic transcription factor Runx2 (140). MSCs from IFN-γR^−/−^ mice show a lower osteogenic differentiation due to lower Runx2 expression. Furthermore, IFN-γ siRNA inhibits osteoblast differentiation from MSCs (139, 141). However, the signaling pathway of IFN-γ in osteoblast differentiation is not clear yet.

#### Effects of IFN-γ on Osteoclasts

**Figure 3:**
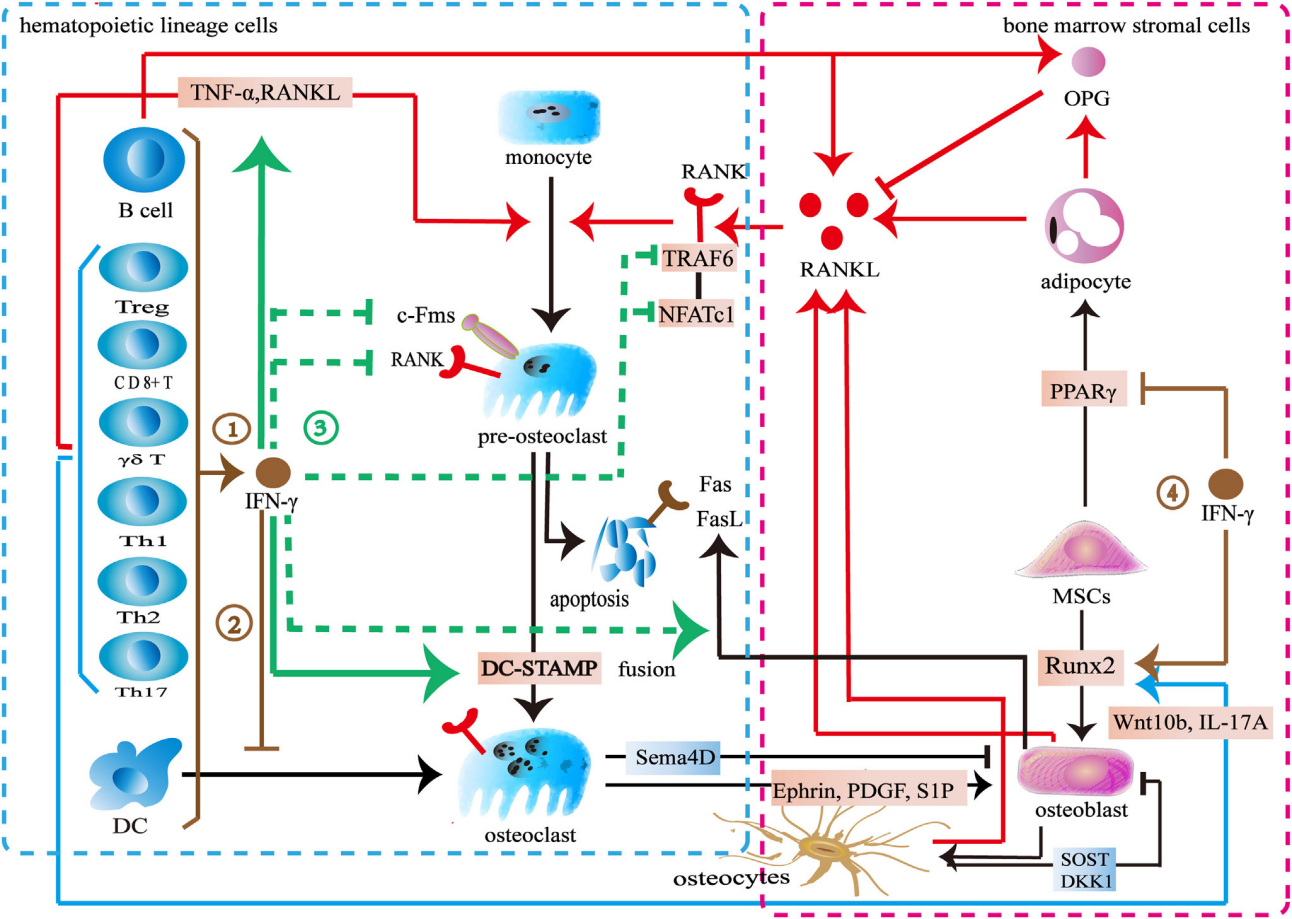
Effect of interferon-gamma (IFN-γ) in osteoimmunology. ① IFN-γ is produced by multiple immune cells including CD4^+^ T cells, CD8^+^ T cells, Treg cells, γδ T cells, B cells, and dendritic cells. ② IFN-γ inhibits the differentiation of DCs into osteoclasts. ③ IFN-γ reduces the expression of c-Fms on the surface of osteoclast precursors to inhibit its proliferation and differentiation. IFN-γ decreases the number of RANK^+^ osteoclast precursors to influence subsequent RANKL-dependent osteoclastogenesis. IFN-γ also increases the degradation of tumor necrosis factor receptor-associated factors 6 (TRAF6) or downregulates NFATc1 in the downstream of RANK–RANKL pathway to inhibit osteoclastogenesis in the early stage of osteoclast differentiation. IFN-γ enhances the apoptosis of osteoclast precursors through FasL. IFN-γ reduces osteoclast formation by directly inhibiting RANK–RANKL pathway, while stimulates the secretion of TNF-α and RANKL by T cells to promote osteoclast formation. IFN-γ promotes the fusion of mononucleate osteoclasts into functional osteoclasts through DC-STAMP. ④ IFN-γ upregulates the osteogenic transcription factor Runx2 and downregulates the adipogenic transcription factor PPARγ.

Previous studies demonstrated an inhibitory effect of IFN-γ on osteoclast differentiation (Figure 3). Various immune cells including B cells, pro-inflammatory M1 macrophages, dendritic cells, and NK cells produce IFN-γ to inhibit osteoclastogenesis (68, 70, 142). Furthermore, both activated and resting T cells secrete IFN-γ to inhibit osteoclast formation. The activated T-cell supernatant inhibits osteoclast differentiation from bone marrow macrophages. Anti-IFN-γ antibodies can block its effect in the culture system (143). The resting T cells also produce IFN-γ to reduce osteoclast formation when co-cultured with peripheral blood monocytes (144). IFN-γ downregulates c-Fms in monocyte-derived osteoclast precursors to reduce RANK. The M-CSF/c-Fms is critical for early osteoclast proliferation and differentiation. The decrease of RANK^+^ osteoclast precursors influences subsequent RANKL-dependent osteoclastogenesis (145). The recognized mechanism of IFN-γ in osteoclastogenesis is to promote TRAF6 degradation in RANKL-induced osteoclast differentiation (26, 146). IFN-γ activates the classical JAK–STAT1 pathway to initiate the ubiquitin-proteasome to increase the degradation of ubiquitin ligase TRAF6. Due to the downregulation of TRAF6, the downstream transcription factors of TRAF6 including NF-κB and JNK are inhibited, therefore, osteoclast formation is reduced (143, 147–149). However, two reports indicated that IFN-γ did not target on TRAF6 (147, 148). IFN-γ inhibits osteoclasts *via* downregulating NFATc1 (148, 149). IFN-γ inhibits osteoclast formation when IFN-γ and RANKL are simultaneously used in bone marrow macrophages or RAW264.7 cells. However, the inhibitory effect is almost completely neutralized after being pretreated with RANKL during a certain period of time (147). RANKL treatment with a short time did not resist the inhibitory effect of IFN-γ on osteoclastogenesis. Furthermore, high concentration RANKL might overly activate both the RANK–RANKL pathway and the RANK intracellular domain (ivvy535–538) to counteract the inhibitory effect (148). Apart from inhibiting osteoclast differentiation, IFN-γ also reduces osteoclast formation by promoting osteoclast apoptosis. IFN-γ inhibits TNF-α-induced osteoclastogenesis by inducing FasL expression in bone marrow cells. IFN-γ promotes osteoclast precursor apoptosis by Fas–FasL interaction (149). IFN-γ might increase FasL expression on osteoblasts by activating the NF-κB pathway to regulate osteoclast apoptosis (150). Furthermore, IFN-γ stimulates osteoblasts to produce NO to promote osteoclast apoptosis (151).

On the other hand, some studies showed that IFN-γ promotes osteoclast formation (Figure 3). IFN-γ promotes osteoclast maturation in the late stage of osteoclast formation. When monocytes/macrophages are differentiated into functional osteoclasts, they need to be matured into functional osteoclasts by fusion. A seven-transmembrane DC-STAMP protein is involved in osteoclast fusion. Miyamoto et al. (152) reported that NFATc1 and c-fos regulated the expression of DC-STAMP. IFN-γ upregulates c-fos and NFATc1 to ultimately increase DC-STAMP expression. Thus, the fusion of osteoclast is enhanced (153). IFN-γ induces osteoclasts to express IDO to inhibit T cells proliferation (146). However, IFN-γ increases MHC class II expression in the antigen-presenting cells to enhance T cells activation. T cells produce more TNF-α and RANKL to promote osteoclast formation (143, 154, 155). Furthermore, IFN-γ promotes multiple immune cells including monocytes and macrophages to secrete CXCL10. CXCL10 induces CD4^+^ T cells to secrete RANKL and TNF-α to promote the differentiation of osteoclasts (156, 157).

These contradictory results may result from the differences in the concentrations of IFN-γ, RANKL, and M-CSF, as well as the exposure time and the stage of osteoclast differentiation. IFN-γ inhibits osteoclast differentiation in a dose-dependent manner. However, low level of IFN-γ does not exert the inhibitory effect in osteoclast and treatment with high level of RANKL antagonizes its inhibitory effect in osteoclast differentiation. Also IFN-γ might potentially lose its inhibitory effect during a certain stage of osteoclasts. The inhibitory effect of IFN-γ seems to be limited to the early stage of osteoclast differentiation. The M-CSF/c-Fms signaling is important for early osteoclast proliferation and differentiation. The RANK–RANKL pathway is also vital for early osteoclast differentiation. IFN-γ downregulates c-Fms and RANK to inhibit osteoclastogenesis in the early stage of proliferation and differentiation. IFN-γ also increases the degradation of TRAF6 or downregulates the NFATc1 in the downstream of RANK–RANKL pathway to inhibit osteoclastogenesis in the early stage of osteoclast differentiation. However, IFN-γ promotes osteoclast maturation by increasing fusion in the late stage of osteoclast formation. Interestingly, there is an overlapped transcription factor. IFN-γ downregulates NFATc1 to inhibit osteoclastogenesis in the early stage of differentiation but upregulates NFATc1 to increase the fusion of osteoclast in the late stage of differentiation. In addition to its direct effect, IFN-γ also indirectly increases osteoclastic factors to promote osteoclast formation by activating T cells (26, 146). However, IFN-γ may reduce T cells proliferation by inducing the IDO in osteoclasts. IFN-γ activates immune response to promote osteoclast formation in the early stage whereas multinucleated osteoclasts inhibit the activation T cells in turn. In summary, the dual effect of IFN-γ in osteoclastogenesis may result from the different role of IFN-γ during different stages of osteoclast differentiation. Furthermore, the differences between direct and indirect actions also lead to the conflicting results.

#### Effects of IFN-γ on Bone Mass

IFN-γ^−/−^ mice do not produce IFN-γ. IFN-γR^−/−^ mice produce IFN-γ but lack the receptors. Both mice show lower bone mass (139, 141, 143, 155). Human T cell lymphotropic virus type 1-Tax transgenic mice (Tax^+^) have the potential to develop osteolytic bone tumors. Due to the absence of IFN-γ, Tax^+^IFN-γ^−/−^ mice show more severe tumor-associated osteolytic destruction and lower bone mass with increased osteoclastogenesis. Administration of IFN-γ recused cancer-induced bone loss in Tax^+^IFN-γ^−/−^ mice (158). Dcir^−/−^ mice are easier to develop ankylosing enthesitis with slightly higher bone mass, while the knockout of IFN-γ counteracts the effect in Dcir^−/−^ mice (137). Duque et al. found IFN-γ increased bone mass in wide-type mice (141).

However, some other studies demonstrated that systematic administration of IFN-γ can reduce bone mass. The clinic trial with recombinant human IFN-γ-1b revealed that IFN-γ promotes bone resorption by functional improvement of osteoclasts (159). Mann et al. found that systemic application of IFN-γ for 8 days led to cancellous bone loss in Sprague-Dawley rats (160). Gao et al. reported that IFN-γ treatment decreased spinal bone mass and increased serum level of C-terminal telopeptides in T cell–replete nude mice and wide-type mice, but not in T cell–nude mice, implicating the importance of T cells (155).

The knockout of IFN-γ or its receptors in animal models induces bone loss. It seems that IFN-γ is necessary to maintain normal bone mass. However, the administration of IFN-γ showed contradictory results. Under a certain medication schedule such as 2,000 or 10,000 IU three times a week for 6 weeks, IFN-γ may increase bone mass (141). However, IFN-γ may cause bone loss in some other schedule such as 1 × 10^6^ IU/kg body weight twice a week for 3 weeks or 1 × 10^6^ IU/kg body weight once a day for 8 days (155, 160). Long-term use of IFN-γ at a low dosage seems to promote bone formation and inhibit osteoclast differentiation at its early stage (141). However, short-term use of IFN-γ at a high dosage seems to act on the late stage of differentiation to promote osteoclast formation (160). Furthermore, short-term use of IFN-γ in a high dosage may activate immune responses especially T cell-mediated immune responses to promote osteoclastogenesis (155). In summary, IFN-γ may be vital to maintain normal bone mass but its dosage, frequency, usage, and course may be important factors. The balance of osteoblasts and osteoclasts, the predominant effect on osteoclasts differentiation, and the activation of immune cells may be the reason behind these contradictory results. Further studies are required to disclose its complex roles in different pathophysiological conditions.

## IFN-γ Mediated Osteoimmunology in Diseases

### Rheumatoid Arthritis

Rheumatoid arthritis is defined as inflammatory arthritis with periarticular and systemic bone loss (161–163). The etiology of RA is not completely clear but it is known that various adaptive immune responses and innate immune responses are involved in. Immune cells including B cells, T cells, NK cells, dendritic cells, macrophages, and immune factors play important roles in RA inflammatory and bone loss (164). Osteoclast activation and osteoblast inhibition lead to bone destruction and bone loss (161). Synovial fibroblasts, activated B cells, and T cells produce RANKL to influence the RANK–RANKL–OPG pathway and regulate osteoclast formation. Furthermore, synovial fibroblasts produce DKK-1 to block the Wnt signaling pathway and inhibit osteoblast formation. Inflammatory cytokines including TNF-α, IL-1, IL-6, IL-17, IL-18, and IL-23 also activate osteoclasts (165, 166).

Interferon-gamma also plays an important role in the development and progression of RA. NK-like T cells, monocytes, T cells, and B cells secret higher levels of IFN-γ in RA (167–171). The high levels of IFN-γ are found in RA plasma, synovial tissue, and synovial fluid (172, 173). With the progression of disease, IFN-γ levels show an upward trend in plasma (173). Cacciapaglia et al. found that the IFN-γ level in mononuclear cells from the peripheral blood was consistent with the serous of RA (174).

Several studies have reported a negative role of IFN-γ in RA. IFN-γ antibodies are a promising advantage to RA patients. Anti-IFN-γ and anti-TNF-α show similar efficacy in alleviating the synovial membrane thickness and clinical indices in RA (175, 176). In animal experiment, IFN-γ antibodies could reduce synovial proliferation, cell infiltration, bone erosion, and cartilage destruction in the joints of onset RA. Meanwhile, systemic administration of IFN-γ increases arthritic scores in the early stage of RA (177). IFN-γ seems to accelerate the onset and development of RA in the early stage. IFN-γ might initiate disease by activating inflammation and immune responses. IFN-γ mobilizes lymphocytes by activating the classic transcription factor STAT1 to promote RA inflammatory (171). Treg cells limit excessive immune responses, whereas IFN-γ derived from B cells inhibits the differentiation of Treg cells thus activating immune response in RA (170). Furthermore, T cells and macrophages activated by IFN-γ produce more RANKL and TNF-α to promote bone erosion. CXCL10 induced by IFN-γ promotes bone resorption by attracting immune cells such as T cells to be involved in RA (156).

Apart from promoting RA progression, some studies highlight a protective role of IFN-γ in RA. Some trials from 1980 to 1995 provided evidence of improvement and benefit. After the administration of IFN-γ, RA clinical symptoms were relieved (178–181). In animal experiment, IFN-γ^−/−^ mice and IFN-γR^−/−^ mice are easier to induce to be a model of RA (182). IFN-γ inhibited anti-CII antibody responses to inhibit type II collagen-induced arthritis (183). By contrast, IFN-γ antibody exacerbated the severity in the late stage of RA (177, 184). IFN-γ inhibits CII-induced T cell proliferation by limiting IgG1 and IgG2b antibody to the autoantigen in RA (185). The high temperature requirement A1 (HTRA1) is a protease and marker for the incidence and severity of RA. IFN-γ initiates p38 MAPK/STAT1 pathway to inhibit HTRA1 in RA joint tissues (166). As a destructive factor in the progression of RA, IL-17 produced by Th17 cells is involved in the RA pathogenesis. IFN-γ inhibits IL-17 production to alleviate RA (184, 186).

Interferon-gamma seems to exert different effects in different stages of RA. In animal experiment, IFN-γ accelerates the onset and development of RA in the early stage. IFN-γ may promote autoimmunity and activate immune response, increasing the level of osteoclastic factors and leading to the development of RA and bone destruction. The indirect stimulating effect of IFN-γ on osteoclasts may be predominant in the early stage of RA. However, IFN-γ seems to have a protective role in RA in the effector phase. The activation of immune systems in the onset of RA can attract more monocytes and macrophages from blood and peripheral tissue into joints to cause massive amplification of osteoclast formation. IFN-γ reduces c-Fms and RANK to inhibit osteoclast formation. IFN-γ also induces TRAF6 degradation or downregulates NFATc1 to inhibit the RANK–RANKL pathway to prevent osteoclast differentiation. In patients with RA, most of studies show administration of IFN-γ is an effective therapy, but some patients fail to display an adequate response. The difference in the genetic and epigenetic factors, the duel effect of IFN-γ in osteoclastogenesis, disease severity, and rate of progression may be the reason for these contradictory results.

### Postmenopausal Osteoporosis

Menopause is a common factor of osteoporosis. Recent researches show that osteoimmunology is involved in the pathogenesis of postmenopausal osteoporosis (187). T-cell activity is increased but B-cells activity is decreased in postmenopausal women (188, 189). Immune cells produce some factors like IL-6, IL-7, IFN-γ, TNF-α, and RANKL to influence the skeletal and immune systems. The multiple cytokines like IL-6, IL-7, and TNF-α enhance the formation or activity of osteoclasts in estrogen deficiency. T cells and B cells produce RANKL to promote osteoclast formation (190, 191). Furthermore, IL-27 has a protective role on bone loss in estrogen deficiency (192). A clinical research showed CD4^+^ T cells secreted a significant lower IFN-γ in women with postmenopausal osteoporosis (187). Whereas two other studies showed no different in IFN-γ in patients with postmenopausal osteoporosis (193, 194). In animal experiment, IFN-γ level in bone marrow was increased after ovariectomy (150). The bone mass in the IFN-γ^−/−^ mice did not significantly reduce after ovariectomy (155). Under estrogen deficiency, IFN-γ promoted the expression of class II transactivator in immune cells to enhance the antigen presentation between macrophages and T cells, which could upregulate TNF-α and RANKL and promote bone resorption (195). It seems at least in animal studies IFN-γ might enhance bone loss in estrogen deficiency through regulating osteoclastogenesis (26). Further study is definitely needed to clarify its pathogenesis.

### Human Immunodeficiency Virus

Acquired immune deficiency syndrome (AIDS) is an acquired defect in cellular immunity associated with infection with the HIV. This syndrome leads to a large destruction of CD4^+^ T cells and immunodeficiency. The patients with AIDS are particularly vulnerable to bone loss and at higher risk for fracture due to HIV infection, antiretroviral therapy, hypogonadism, lower nutritional status, etc. (86). B cells produce more RANKL and less OPG to promote osteoclast formation in AIDS. After antiretroviral therapy, immune cells including T cells, B cells, and macrophages produce high levels of RANKL and TNF-α to activate osteoclasts (196). The serum level of IFN-γ is lower in exposed-uninfected individuals than that in HIV-unexposed individuals, but there is no data about the main source of IFN-γ. The lower level of IFN-γ might reduce antigen presentation and HLA class II expression to defend HIV infection (197). Some studies have shown IFN-γ inhibits HIV infection but the mechanism remains unknown (198, 199). Another study showed that IFN-γ reduced the expression of CXCR-4 and CCR-5 in monocyte surfaces to inhibit HIV replication (200). IFN-γ inhibits HIV-1 replication stimulated by RANKL (201). The gp120-stimulated T cells secrete RANKL to enhance osteoclast formation, which is an important reason for HIV-related bone loss. IFN-γ might inhibit RANKL-mediated osteoclast formation by promoting TRAF6 degradation in AIDS (201). The positive effect of IFN-γ on HIV infection and HIV-related bone loss make it a promising agent for clinical application.

## Conclusion and Prospect

Over the past decade, osteoimmunology has been proposed as a new interdiscipline to understand the relationship between the skeletal and the immune systems. The effects of various immune factors on bone metabolism have led to the investigations of their clinical application. IFN-γ plays an important role in bone homeostasis *via* activation of complex signaling pathways. IFN-γ potentially promotes osteoblastogenesis but inhibits bone marrow adipocyte formation. However, IFN-γ plays conflicting roles in osteoclastogenesis. Its functions are dependent on the balance of direct or indirect effect, as well as the stage of osteoclast differentiation. IFN-γ inhibits the early differentiation of osteoclasts by targeting the RANK–RANKL pathway, whereas it promotes the fusion of mononucleated osteoclasts in the late stage of osteoclast formation. IFN-γ indirectly increases osteoclastic factors by activating immune responses. Under a condition of complete loss of IFN-γ signaling, normal bone mass cannot be maintained. However, the administration of IFN-γ may cause bone loss or inverse. It depends on the dosage, frequency, usage, and course. Furthermore, the pathogenesis, the disease severity, and rate of progression influence the effect of IFN-γ on immune-mediated bone diseases.

Future research should focus on the molecular pathways and conditions related to the different functions of IFN-γ in osteoimmunology. IFN-γ plays dual effect in osteoclastogenesis. Most studies showed that IFN-γ acts on multiple sites of RANK–RANKL pathway including RANK, TRAF6, and NFATc1. It is urgent to find out the key initial site. NFATc1 is an overlapped transcription factor. It is worth to investigate its role in the dual effect of IFN-γ in osteoclast formation. Second, we need to determine the appropriate dosage, frequency, usage, and course to apply IFN-γ in immune-mediated bone diseases. It is important to explore the effect of IFN-γ according to the pathological mechanisms of the disease. Regarding to RA, IFN-γ seems to exert different effects in different stages. Therefore, it may be important to find a suit appropriate time to use and explore the role of IFN-γ in the skeletal and immune systems in RA.

## Author Contributions

XY provided the conception of the manuscript. MT and LT were contributed to writing of the manuscript. MT and GL provided the figure.

## Conflict of Interest Statement

The authors declare that the research was conducted in the absence of any commercial or financial relationships that could be construed as a potential conflict of interest.
